# Lethal Waterhouse**–**Friderichsen syndrome caused by *Capnocytophaga canimorsus* in an asplenic patient

**DOI:** 10.1186/s12879-022-07590-1

**Published:** 2022-08-17

**Authors:** Franziska Schuler, Jan-Sören Padberg, Carsten Hullermann, Philipp Kümpers, Johannes Lepper, Miriam Schulte, Andreas Uekötter, Frieder Schaumburg, Barbara C. Kahl

**Affiliations:** 1 Institute of Medical Microbiology, University Hospital Münster, Münster, Germany; 2 Department of Cardiology, University Hospital Münster, Münster, Germany; 3 Department of Medicine D, Division of General Internal Medicine, Nephrology, and Rheumatology, University Hospital Münster, Münster, Germany; 4 Gerhard Domagk Institute of Pathology, University Hospital Münster, Münster, Germany; 5 MVZ Labor Münster Hafenweg GmbH, Münster, Germany

**Keywords:** *Capnocytophaga canimorsus*, Dog bite, Waterhouse–Friderichsen Syndrome, sepsis, Case report

## Abstract

**Background:**

*Capnocytophaga canimorsus*, a Gram-negative rod, belongs to the Flavobacteriaceae family and colonizes the oropharynx of dogs and cats. Infections with *C. canimorsus* are rare and can induce a systemic infection with a severe course of the disease. So far, only five case reports of *C. canimorsus* infections associated with Waterhouse–Friderichsen Syndrome (WFS) have been reported with only two of the patients having a history of splenectomy.

**Case presentation:**

Here, we report a fatal case of WFS due to *C. canimorsus* bacteremia and mycetal superinfection in a 61-year-old female asplenic patient. Despite extensive therapy including mechanical ventilation, antibiotic coverage with meropenem, systemic corticosteroids medication, vasopressor therapy, continuous renal replacement therapy, therapeutic plasma exchange, multiple transfusions of blood products and implantation of a veno-arterial extracorporeal membrane oxygenation the patient died 10 days after a dog bite. The autopsy showed bilateral hemorrhagic necrosis of the adrenal cortex and septic embolism to heart, kidneys, and liver. Diagnosis of *C. canimorsus* was prolonged due to the fastidious growth of the bacteria.

**Conclusions:**

The occurrence of a severe sepsis after dog bite should always urge the attending physician to consider *C. canimorsus* as the disease-causing pathogen. A therapeutic regimen covering *C. canimorsus* such as aminopenicillins or carbapenems should be chosen. However, despite maximum therapy, the prognosis of *C. canimorsus*-induced septic shock remains very poor. Asplenic or otherwise immunocompromised patients are at higher risk for a severe course of disease and should avoid exposure to dogs and cats and consider antibiotic prophylaxis after animal bite.

## Background

*Capnocytophaga canimorsus* are Gram-negative rods that belong to the family Flavobacteriaceae and are members of the microflora in the oral mucosa of dogs and cats [1]. The oral carriage of. *C. canimorsus* in healthy dogs ranges from 3 to 74% [2]. The microscopic appearance in combination with the history of a dog bite give a strong hint for *C. canimorsus* infection.

Infections with *C. canimorsus* are rare and can induce a systemic infection with a fulminant course of the disease. The total number of documented cases of *C. canimorsus* infections from 1990 to 2014 is 292 with a Case-fatality rate about 24% [2]. Splenectomy and alcohol abuse are common predisposing factors, but up to 40% of patients have no obvious risk factor, implying that *C. canimorsus* cannot solely be considered an opportunistic pathogen [2–5]. A history of dog scratching or biting, but also licking of pre-existing wounds is common [2]. However, the infection has also been reported among patients with no evidence of a skin injury [6]. Only five case reports of *C. canimorsus* infection associated with Waterhouse–Friderichsen Syndrome (WFS) have been reported yet [7–11]. Mortality was extremely high (100%) with only two of the patients having a history of splenectomy.

*C. canimorsus* are surrounded by capsular polysaccharides, which affect the host–pathogen interaction leading to resistance to the innate immune systems [12–14]. The lack of adequate B-1a cells and the absence of splenic marginal zone B cells seem to play a major role in the poor response to and clearance of encapsulated organisms in asplenic persons [15, 16]. *C. canimorsus* strains belonging to the capsular serovars A, B and C are responsible for most of the human infections and are present in different geographic areas, despite constituting only a minority of isolates cultured from dogs. There is controversy about the role of strains belonging to capsular serovars D–K, which are considered less virulent especially when infecting asplenic or otherwise immunocompromised persons [12, 14].

## Case presentation

We report a fatal case of WFS due to bacteremia with *C. canimorsus* in a 61-year-old asplenic female.

**Fig. 1:**
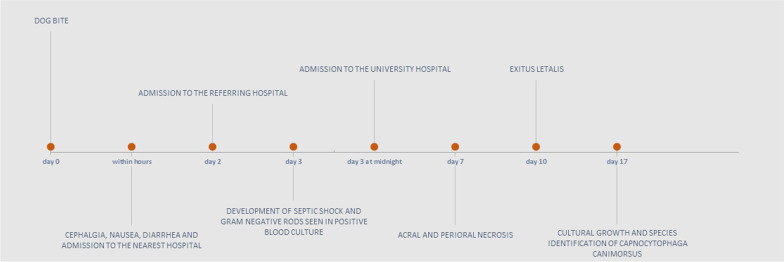
Timeline of the episode of care

The patient was bitten on the third finger of the right hand by her own dog, and developed cephalgia, nausea and diarrhea on the same day. She was admitted to the nearest hospital (Fig. 1) and covered with piperacillin/tazobactam (4 g/0.5 g tid) and gentamicin (320 mg qd).

The history of the patient revealed a systemic Lupus erythematodes (without immunosuppressive therapy), arterial hypertension, mitral valve regurgitation, angiodysplasia of the colon with recurrent hemorrhages, polyarthrosis and fibromyalgia. The patient had been splenectomised in the 1980s for an unknown reason.

Within 24 h, a massive platelet drop and an INR elevation in the sense of disseminated intravascular coagulopathy (DIC) occurred. Similarly, purpura developed, especially in the face and upper trunk. Because of the purpura, WFS was suspected and antibiotic therapy was changed to ceftriaxone and ampicillin after obtaining microbiological samples.

Blood cultures (BD, Heidelberg) turned positive 16.5 h after incubation in an automated blood culture system (Bactec, BD, Heidelberg) and revealed Gram-negative rods by Gram staining. Cultures remained negative despite three repeated attempts every second day on Columbia blood agar and Chocolate agar (incubation at 37 °C with 5% CO_2_; both agars from Oxoid, Wesel, Germany), Schaedler agar (anaerobic incubation at 37 °C; Oxoid, Wesel, Germany) and MacConkey agar (aerobic incubation at 37 °C; Oxoid, Wesel, Germany). Additionally, at admission to the hospital, the patient was tested positive for SARS-CoV-2 on a rapid antigen diagnostic test (Ag-RDT), which was not verified by SARS-CoV-2 nucleic acid amplification.

Subsequently, a progressive decrease in vigilance occurred, whereupon the patient was transferred to the intensive care unit of the University Hospital Münster, a tertiary care hospital.

On arrival, she was in septic shock with ubiquitous purpura fulminans. She had a massive cytokine release syndrome (IL-6: 140,000 ng/mL), procalcitonin of 138 ng/mL, type A lactic acidosis, and severe DIC. The patient received extensive therapy, including mechanical ventilation, antibiotic coverage with meropenem (2 g qid as prolonged infusion), clindamycin (900 mg tid) and ampicillin (3 g qid), systemic corticosteroids (methylprednisolone 500 mg tid, 250 mg bid, 250 mg qd, 125 mg qd), vasopressor therapy, continuous renal replacement therapy (with additional Seraph^®^ 100 Microbind^®^ Affinity Blood Filter, ExThera Medical). Patients with acquired protein C deficiency may develop purpura fulminans. Therefore, during plasmapheresis in total around 40 units of Fresh Frozen Plasma had been administered as a source of protein C. Septic cardiomyopathy was managed by implantation of a veno-arterial extracorporeal membrane oxygenation (vaECMO).

**Fig. 2:**
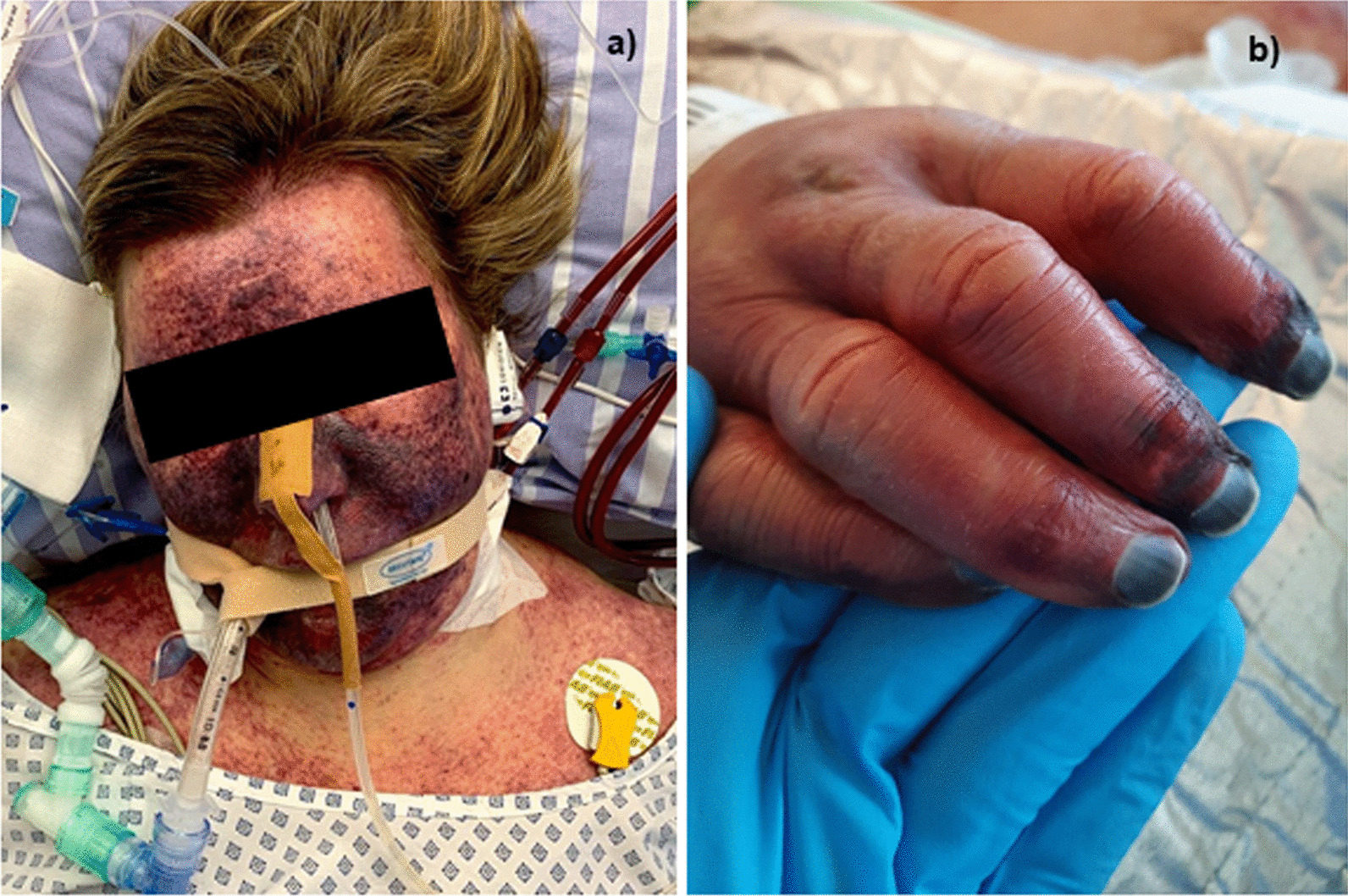
Clinical presentation of Waterhouse–Friderichsen syndrome by *Capnocytophaga canimorsus*. **a** The patient presented with facial purpura as well as **b** perioral and acral necrosis

While the patient initially stabilized clinically, her condition worsened quickly 5 days after admission. In the course of the disease, the patient developed dry perioral and acral necroses on the right and left hand and a paralytic ileus (Fig. 2). She finally died 10 days after the dog bite due to refractory septic shock with WFS.

Blood cultures collected on the day the patient died were positive for *Candida albicans*, which was interpreted as a mycetal superinfection most likely due to transmigration of Candida due to the paralytic ileus.

**Fig. 3:**
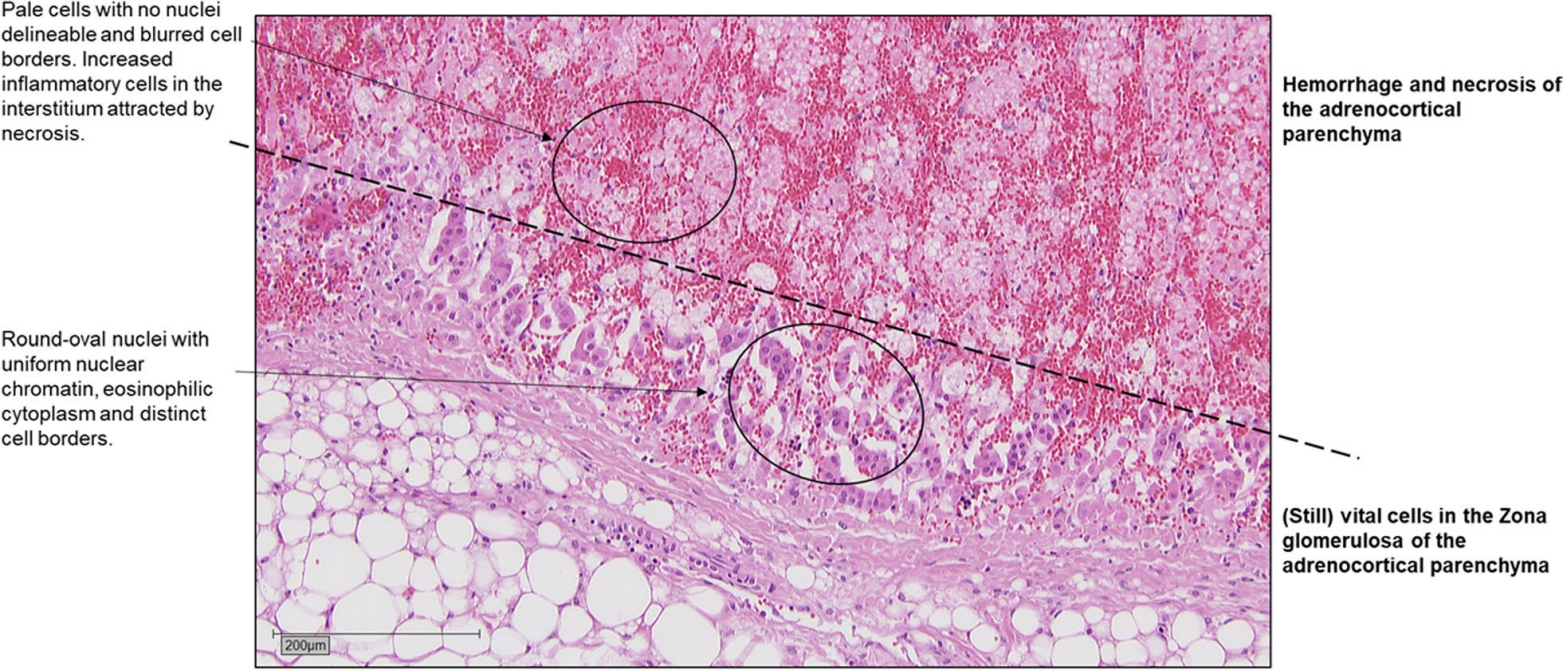
Hemorrhage of the adrenal gland with areas of necrosis using hematoxylin–eosin stain. Microscopy images were taken with a Leica DM5500 B microscope (Leica, Wetzlar, HE, Germany; 20 × magnification), images were captured on a CCD camera and adjusted with DISKUS program

The autopsy showed bilateral hemorrhagic necrosis of the adrenal cortex (Fig. 3), septic embolism to heart, kidneys, and liver with mycetal superinfection. Consequently, the patient was diagnosed with WFS. A minor, superficial skin defect in the area of the right middle finger after the dog bite classified as grade I using the Rueff classification of animal bites was considered as the port of entry for the pathogen [17].

**Fig. 4:**
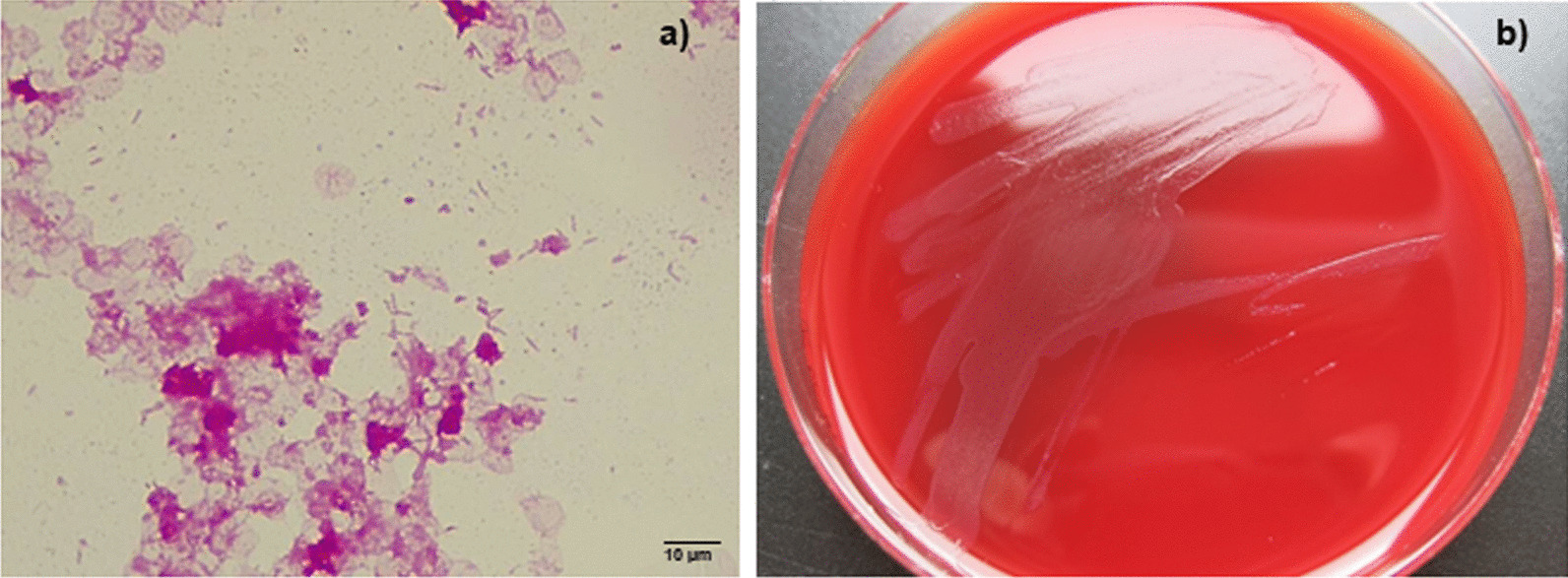
Culture of *Capnocytophaga canimorsus*. **a** Gram-staining of *C. canimorsus* from blood culture showing subtle Gram-negative rods. The image was taken with a Leica CM750 microscope (1000 × magnification) and captured with a camera attached to the microscope (Leica DMshare software). **b** Growth of *C. canimorsus* on Columbia blood agar revealing small, greyish, sharply defined colonies after 48 h

Post-mortem, blood culture bottles obtained at the referring hospital were sent to our microbiology laboratory for detailed analysis. We confirmed long, thin, Gram-negative rods after Gram staining (Fig. 4). Within 48 h, small, greyish, sharply defined colonies were visible on BBL Columbia blood agar with 5% sheep blood (BD, Heidelberg, Germany) after incubation at 37 °C under 5% CO_2_. Identification based on MALDI-TOF (Bruker, Bremen, Germany) revealed *C. canimorsus.* Antibiotic susceptibility testing was performed using Epsilometric tests (Etest^®^, biomérieux, France) in accordance to the European Committee on Antimicrobial Susceptibility Testing (EUCAST; version 10.0) and minimal inhibitory concentrations (MIC) were interpreted using non-species related clinical breakpoints. As there was no growth on standard agar plates for antibiotic susceptibility testing (Mueller Hinton agar or Mueller Hinton agar with horse blood), BBL Columbia blood agar with 5% sheep blood was used. The isolate was susceptible to penicillin (MIC: 0.047 mg/L), ceftriaxone (MIC: 0.19 mg/L) and meropenem (MIC: 0.004 mg/L).

**Fig. 5:**
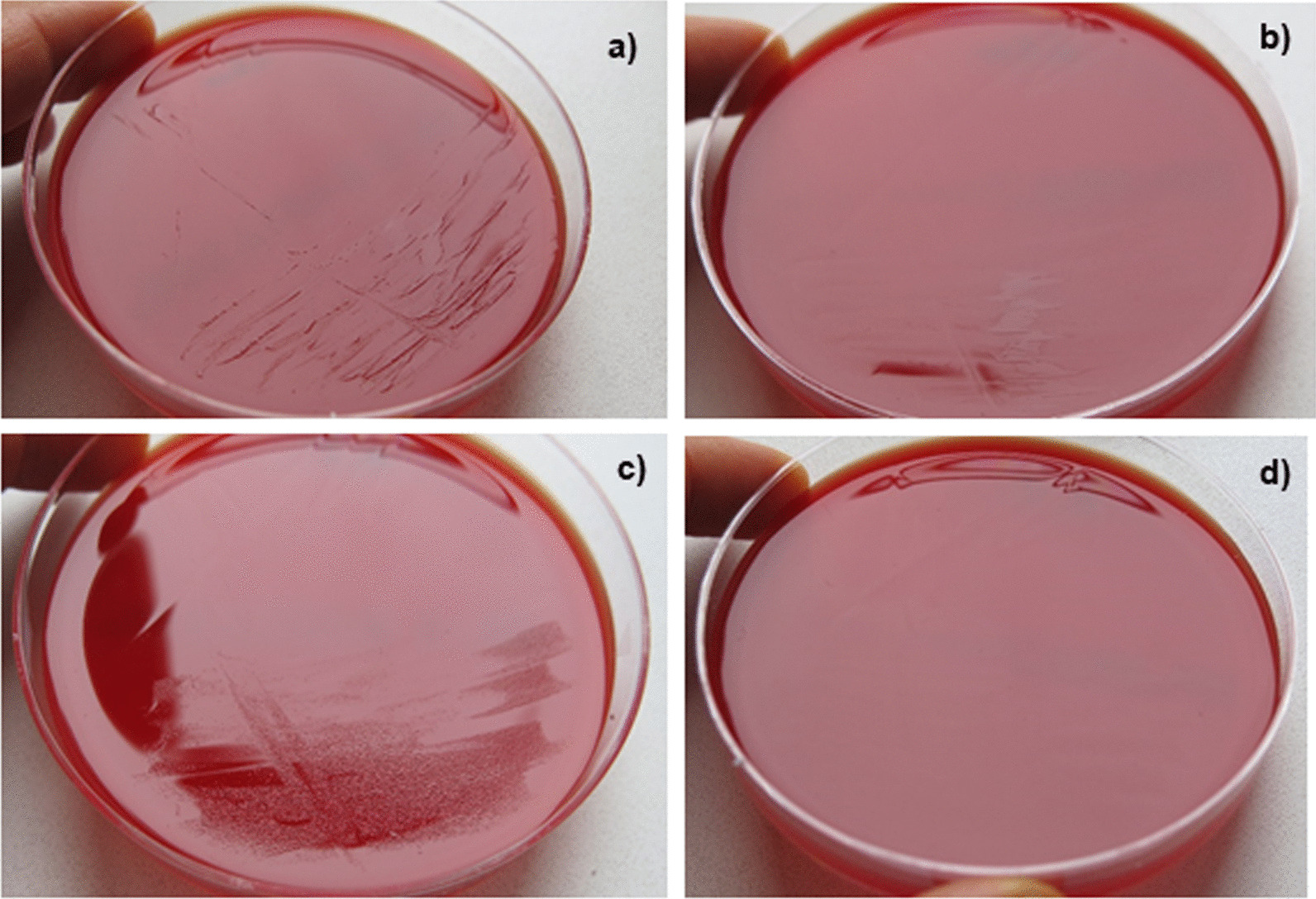
Growth of *Capnocytophaga canimorsus* on Columbia blood agar of two different manufacturers. Positive blood cultures were inoculated on Columbia Agar with 5% sheep blood from (**a**) BD and (**b**) Oxoid. The inoculum of pure cultures was standardized (McFarland 0.5) and sub-cultured on Columbia blood agar from (**c**) BD and (**d**) Oxoid. The comparison of the media from two manufacturers revealed a (**b**) weaker or (**d**) absent growth on agar plates from Oxoid

In order to confirm that the unsuccessful culture in the referring laboratory was due to different manufacturers of Columbia blood agar, we compared the growth of *C. capnocytophaga* on Columbia blood agar using our standard media and the standard media from the referring laboratory. Varying results (weaker respectively absent growth on agar plates from Oxoid) were confirmed (Fig. 5). We were unable to identify the reason for the discrepant growth on agar plates from different manufacturers. The classical formula of both agar plates showed the same composition of ingredients.

## Discussion and conclusions

Early in the 20th century, Waterhouse and Friderichsen described the first cases of bacterial sepsis in children associated with bilateral adrenal hemorrhage, a diseases entity, which was then named as “Waterhouse–Friederichsen Syndrome” [18, 19]. However, as more WFS cases caused by various bacterial and also viral infections were reported, such cases not only included bacterial or viral infections associated with bilateral adrenal hemorrhage, but also with adrenal insufficiency, which mostly but not necessarily were associated with bilateral adrenal hemorrahge, leading to a description of WFS in a broader clinical meaning.

The initial presenting complaints for patients with the WFS usually include a diversity of nonspecific, vague symptoms such as headache, fever, weakness, fatigue, abdominal or flank pain, nausea or vomiting, confusion, or disorientation. These symptoms are usually abrupt in their onset. More than 75% of patients develop a generalized rash, that usually begins as a pink, maculopapular eruption on the extremities [20]. Petechiae are present in approximately 50–60% of patients and often appear initially on the ankles and wrists and in the axillae and may spread to any part of the body (including the conjunctiva) [21]. Purpura fulminans is characterized by the acute onset of cutaneous hermorrhage and necrosis due to vascular thrombosis and DIC [22].

To our knowledge the case fatality rate of WFS has not been assessed systematically yet. In meningococcal infections, 44% ot the patients who present with purpura have a lethal outcome [23]but no correlation to bilateral adrenal haemorrhage has been studied. The diagnosis of *C. canimorsus* is often delayed because sub-cultures from primary blood culture media to solid plating media may be unsuccessful, even when many organisms were seen in large numbers after Gram staining [24]. Noteworthy, in our case the cultivation of bacteria from the initial positive blood culture did not succeed in the referring laboratory. The only obvious difference in diagnostics was the different manufacturer of the solid media (Oxoid vs. BD). Additionally, the cultivation of *C. canimorsus* in our laboratory from the initial positive blood culture was still possible even 13 days after the blood culture became positive indicating that the bacteria were still viable after almost two weeks.

In a study by Dusch et al. from 1994, the authors observed that three different strains of *C. canimorsus* did not grow on BBL Columbia agar (BD), which worked well in our case [24]. However, the formulation of the agar might have changed since then. In contrast, the bacteria did not grow on the agar from the other manufacturer. Thus, media formulation might play a critical role for this fastidious organism. Therefore, we recommend, if sub-cultures from positive blood cultures on solid media (e.g. Columbia blood agar, chocolate agar) reveal no growth, agar plates from different manufactures should be used.

Just as with our patient, a false-positive Ag-RDT in a patient with lethal *C. canimorsus* bacteremia has been recently published by Meyer et al. [25]. During the acute phase of hyper-inflammation and cytokine storm, false positive Ag-RDT might therefore cause fixation errors during the SARS-CoV-2 pandemic [25].

Within 24 h after admission to the first hospital, the calculated therapy was changed from tazobactam-piperacillin and gentamicin to ampicillin and ceftriaxone. Infections caused by dog bites are likely to be caused by both aerobic and anaerobic bacteria. Although we consider *C. canimorsus* likely to be the sole causative agent of severe sepsis, an undetected anaerobic bacterium would not have been covered by ampicillin and ceftriaxone.

While there are reports of ß-lactamase producing *Capnocytophaga* strains as reviewed by Jolivet-Gougeon et al., the initial treatment of this disease could have failed by using ceftriaxone and ampicillin [26]. However, susceptibility testing revealed an overall susceptible isolate.

In conclusion, the occurrence of a sepsis after dog bite should always urge the attending physician to consider *C. canimorsus* as the disease causing pathogen. A therapeutic regimen covering *C. canimorsus* such as aminopenicillins or carbapenems should be chosen. However, despite maximum therapy, the prognosis of *C. canimorsus*-induced septic shock still remains very poor. Immunocompromised patients are at higher risk for a severe course of disease and should avoid exposure to dogs and cats.

Antibiotic prophylaxis is discussed critically for patients who present for evaluation of bite injury in the absence of signs or symptoms of infection. The risk for severe infections in asplenic patients has not been assessed systemically yet. In general, antibiotic prophylaxis for patients with clinically uninfected wounds is suggested for wounds on the hand(s), face or genital area, wounds in close proximity to a bone or joint, wounds in immunocompromised or asplenic hosts and lacerations undergoing primary closure or wounds requiring surgical repair [27–29]. According to this data our patient showed at least two risk factors for severe infection (asplenia and bite into her hand). Therefore patients at risk should be educated about an early administration of antibiotics. An antimicrobial therapy against both aerobic and anaerobic bacteria such as amoxicillin-clavulanic acid should be chosen [29].

## Data Availability

The datasets used and analysed during the current study are available from the corresponding author on reasonable request.

## Abbreviations

Ag-RDT: Rapid antigen diagnostic test
*Capnocytophaga canimorsus*: *C. canimorsus*
EUCAST: European Committee on Antimicrobial Susceptibility Testing
MIC: Minimal inhibitory concentration
WFS: Waterhouse–Friderichsen syndrome
DIC: Disseminated intravascular coagulopathy
qd: Once a day
bid: Twice a day
tid: Three times a day
qid: Four times a day
